# Integrated single‐cell RNA sequencing analyses suggest developmental paths of cancer‐associated fibroblasts with gene expression dynamics

**DOI:** 10.1002/ctm2.487

**Published:** 2021-07-19

**Authors:** Hee Chul Chung, Eun Jeong Cho, Hyeonjin Lee, Won‐Kyung Kim, Ji‐Hye Oh, Seok‐Hyung Kim, Dakeun Lee, Chang Ohk Sung

**Affiliations:** ^1^ Center for Cancer Genome Discovery Asan Institute for Life Sciences Asan Medical Center Songpa‐gu Seoul Republic of Korea; ^2^ Department of Pathology Asan Medical Center University of Ulsan College of Medicine Songpa‐gu Seoul Republic of Korea; ^3^ Department of Medical Science Asan Medical Institute of Convergence Science and Technology Asan Medical Center University of Ulsan College of Medicine Seoul Republic of Korea; ^4^ Department of Pathology and Translational Genomics Samsung Medical Center Sungkyunkwan University School of Medicine Seoul Republic of Korea; ^5^ Department of Pathology Ajou University School of Medicine Suwon Republic of Korea

Dear Editor,

The origin and the phenotypic heterogeneity of cancer‐associated fibroblasts (CAFs) are suggested by various models, but not completely understood.^1^, ^2^, ^3^ We used six publicly available single‐cell RNA sequencing (scRNA‐seq) datasets of five cancer types (except breast cancer) on CAFs and corresponding normal fibroblasts (NFs) (Figure 1A)^4^, ^5^, ^6^ and established a comprehensive model for CAF development and gene expression dynamics over time. The fibroblast fraction constituted less than 10% of all cellular components in each dataset (Figure 1B). This relatively low fraction may be ascribed to our two‐step, strict procedure for defining fibroblasts.

**FIGURE 1:**
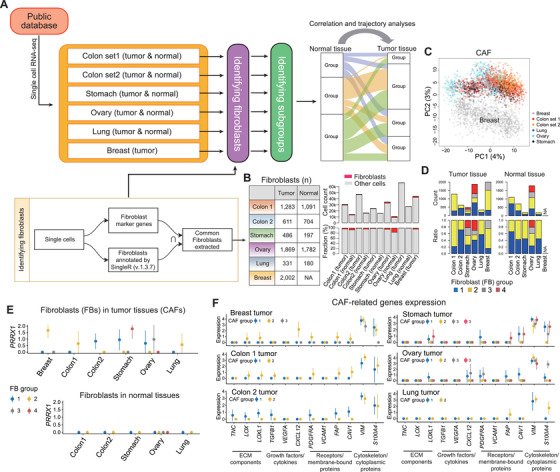
Overall study design and identification of perpetually activated cancer‐associated fibroblast (paCAF) groups. (A) Single‐cell RNA sequencing dataset used in this study and the overview of the procedure for fibroblast identification. (B) Summary of fibroblasts identified in each dataset. (C) Principal component analysis (PCA) demonstrating the clustering of CAFs according to the organs, indicating that the batch effect was unremarkable. Based on global gene expression patterns, only CAFs in breast cancer were found to be distinctively different from the CAFs from other organs. (D) Number and proportion of fibroblast clusters identified in each dataset using K‐means clustering. (E) *PRRX1* expression (median) in each fibroblast group. High *PRRX1* expression was observed only in certain fibroblast groups from each cancer tissue, whereas it was not observed in any of the fibroblast groups from normal tissues. (F) Expression of known CAF‐related genes (median) in CAF clusters. High expression of these genes was observed only in the CAF cluster(s) with high *PRRX1* activity. Therefore, these CAFs were defined as paCAFs in this study

Based on the global gene expression patterns, breast cancer CAFs were markedly different from CAFs from other organs (Figure 1C). K‐means clustering with optimal k number calculated using the sum of squared error for each sample and subsequent principal component analyses revealed the presence of several CAF and NF clusters (Figure S1). Based on the recent discovery of *PRRX1* as a critical regulator of the fibroblast‐specific key transcriptional network,^7^ we examined *PRRX1* expression in each cluster. None of the NFs exhibited *PRRX1* activity, whereas certain CAF clusters showed significantly high *PRRX1* expression (Figure 1E). Furthermore, the known CAF‐related genes in various functional categories were upregulated only in the CAF clusters with high *PRRX1* activity (Figure 1F). Thus, we labeled these CAFs as “perpetually activated CAFs” (paCAFs), which were constituted approximately 50%–80% of all CAFs in each dataset (Figure 1D).

Bone marrow‐derived mesenchymal stem cells (BM‐MSCs) or local tissue‐resident (tr)‐fibroblasts were suggested as the primary source of CAFs; therefore, we examined BM‐MSC markers.^2^, ^8^ CAFs generally express higher levels of BM‐MSC markers than NFs (Figure S2). Subgroup analysis revealed that the CAF clusters with higher BM‐MSC marker expression only comprised paCAFs (Figures 2A and 2B). Meanwhile, since one NF cluster from each set also showed high BM‐MSC marker expression, we named these “tr‐MSC‐like fibroblasts” (tr‐MSCFs) (Figures 2C and 2D). We confirmed that both tr‐MSCFs and paCAFs showed lack of expression of hematopoietic stem cell markers (Figure S3), implying that they were derived from BM‐MSCs. Owing to the relatively low transcriptional activities in the remaining NFs (Figure S4), they were named tr‐resting fibroblasts (tr‐RFs). These cells were considered terminally differentiated mature tissue fibroblasts with no phenotypical plasticity. However, they may have originated from tr‐MSCFs, at least partly. In the paCAF group investigation, one paCAF cluster showed a myofibroblastic (my)CAF signature (Figures 2E and 2F), whereas the other showed an inflammatory (i)CAF signature.^9^ Further subgroup analysis using unsupervised k‐means clustering on colon and lung cancer dataset paCAFs revealed two subclusters with a myCAF or iCAF signature (Figures 2G‐2I, and S5A).

**FIGURE 2:**
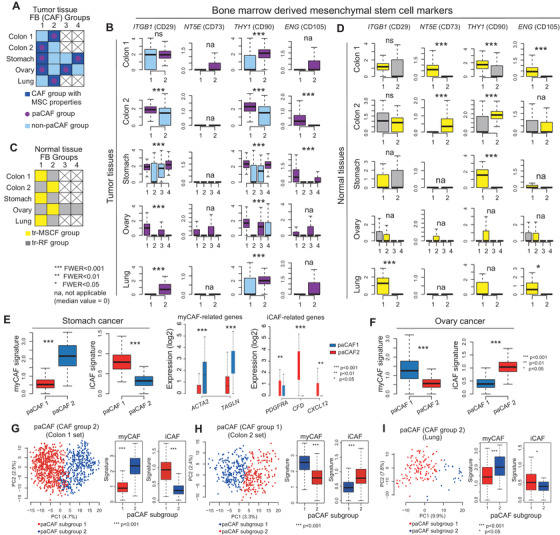
Characteristics of cancer‐associated fibroblasts (CAFs) and their heterogeneous subtypes. (A and B) Assessment of bone marrow‐derived mesenchymal stem cell (BM‐MSC) markers in CAF clusters. The CAF clusters expressing high levels of BM‐MSC markers only comprised perpetually activated cancer‐associated fibroblasts (paCAFs). (C and D) Assessment of BM‐MSC markers in normal fibroblast (NF) groups. One NF cluster in each organ exhibited high expression of BM‐MSC markers, named tissue resident‐MSC‐like fibroblasts (tr‐MSCFs). The remaining fibroblasts among NFs were named tr‐resting fibroblasts (tr‐RFs). (E and F) One of the CAF cluster represented the myofibroblastic (my)CAF signature, whereas the other group represented the inflammatory (i)CAF signature in both stomach and ovarian cancers (Wilcoxon rank‐sum test). myCAFs exhibited high *ACTA2* and *TAGLN* expression, whereas iCAFs exhibited high *PDGFRA*, *CFD*, and *CXCL12* expression. (G‐I) The subclusters of the paCAF group of colon set 1, colon set 2, and lung set identified by further subgroup analyses also represented the myCAF or iCAF signature

Next, to trace the origin of paCAFs, we performed all possible pairwise correlation analyses. In colon and lung cancers, paCAFs showed exclusive correlation with tr‐MSCFs, whereas tr‐RFs were associated with non‐paCAFs (Figures 3A and S6A). Trajectory inference analyses indicated the progressive differentiation of tr‐MSCFs into paCAFs (Figure 3B). This suggested that the paCAFs can be originated from tr‐MSCFs in the adjacent normal tissue, although the split trajectories in these datasets also suggested that some paCAFs may have originated from tr‐RFs or non‐paCAFs. Analyses of the transition between the two paCAF subclusters revealed a significantly longer pseudotime in myCAFs than in iCAFs, indicating serial gene expression transition (Figure 3C). RNA velocity analysis also supported the paths of the tr‐MSCF‐iCAF‐myCAF axis (Figure 3D). These findings were reproduced in independent “colon set 2″ (Figure 3E). In lung cancer, myCAFs and iCAFs showed narrow pseudotime zones, and the transition from iCAFs to myCAFs was unremarkable, probably owing to the low number of fibroblasts (Figure S6B). The tr‐MSCFs were also exclusively correlated with paCAFs in the stomach and ovaries (Figure 3F). In addition, trajectory analysis revealed that tr‐MSCFs, iCAFs, and myCAFs were on a continuous progression with increasing pseudotime in stomach cancer (Figures 3G‐3I), indicating that tr‐MSCFs are first transformed into iCAFs and then to myCAFs (Figure 3J). In ovarian cancer, two distinct paths of tr‐MSCF progression, related to the peritoneum and omentum, were observed (Figure 3K). The iCAFs in ovarian cancer were located halfway along the trajectory from the tr‐MSCFs to myCAFs, and the tr‐MSCFs and iCAFs were mixed in the front end of the continuous line of increasing pseudotime (Figures 3L and 3M), suggesting two different routes of development: (1) tr‐MSCFs to myCAFs via iCAFs, or (2) direct transition of tr‐MSCFs to myCAFs (Figure 3N). We validated the trajectory analyses of CAFs using Slingshot (Figure S6C). The gene expression profiles of tr‐MSCFs were more similar to those of iCAFs compared with myCAFs (Figures 3O and S7A).

**FIGURE 3:**
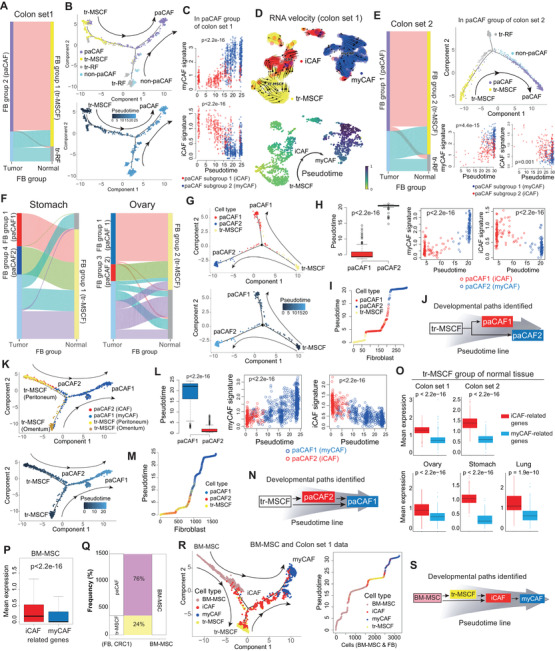
Developmental paths of perpetually activated cancer‐associated fibroblasts (paCAFs). (A) With respect to global gene expression, paCAFs are almost exclusively correlated with tissue resident‐mesenchymal stem cell‐like fibroblasts (tr‐MSCFs) among neighboring normal fibroblasts (NFs). Meanwhile, non‐paCAFs are closely correlated with tr‐resting fibroblasts (tr‐RFs) (Spearman's correlation test). (B) Trajectory and pseudotime analyses indicated the dynamic changes in tr‐MSCFs, iCAFs, and myCAFs. (C) The myCAF signature had a longer pseudotime than the iCAF signature. (D) RNA velocity analysis suggested the serial path of tr‐MSCF–iCAF–myCAF in the pseudotime line. (E) Correlation, trajectory, and pseudotime analyses performed in independent colon set 2, which yielded similar results. (F) With respect to global gene expression in the stomach and ovary, paCAFs were also exclusively correlated with tr‐MSCFs among neighboring NFs (Spearman's correlation test). The trajectory and pseudotime analyses revealed dynamic changes in tr‐MSCFs, iCAFs, and myCAFs in the stomach set (G‐J) and ovary set (K‐N). (O) tr‐MSCFs showed higher levels of iCAF signature than myCAF signature levels in all cancer types (Wilcoxon rank‐sum test). (P) BM‐MSCs also showed higher levels of iCAF signature than myCAF signature levels. (Q) The best correlated BM‐MSC with fibroblasts between tr‐MSCFs and paCAFs (colon set 1) (Spearman's correlation test). (R and S) Trajectory analysis among BM‐MSCF, tr‐MSCF, and paCAF indicating the potential developmental paths of paCAF

In addition, using publicly available BM‐MSC data (GSE147287),^10^ we identified that BM‐MSCs, like tr‐MSCFs, showed higher iCAF‐related genes expressions (Figure 3P). Correlation analysis between BM‐MSCs and the fibroblasts (colon set1) showed that subset of BM‐MSCs were best correlated with tr‐MSCFs (Figure 3Q) and were indicated as a source of tr‐MSCFs as well as paCAFs by trajectory analysis (Figure 3R) after batch correction (Figure S7B), which suggested the developmental paths of CAFs, starting from BM‐MSCs to myCAFs (Figure 3S). Additionally, we extracted "genuine CAF signature” based on our model in colon cancer and was associated with poorer prognosis independent of the stage (Figure S8 and Table S1).

Collectively, during cancer development, tr‐MSCFs migrate to the cancer site and transform into paCAFs (first to iCAFs and then to myCAFs). BM‐MSCs are simultaneously recruited to cancer sites in response to signaling cues from cancer cells and then transform into iCAFs, or subsequently into myCAFs. Meanwhile, with the increase in neoplasms, the dominant cancer niche gradually engulfs the tr‐RF territory. As tr‐RFs are terminally differentiated mature fibroblasts, they do not transform into paCAFs at the cancer site and constitute a group of NF‐like cells among CAFs (Figure 4).

**FIGURE 4:**
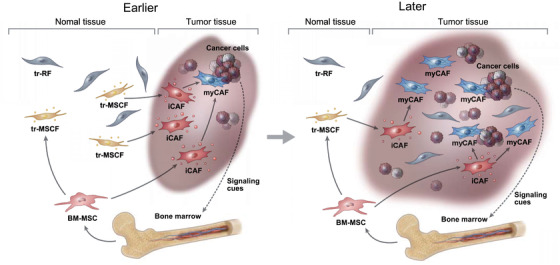
Summary of the suggested developmental paths of perpetually activated cancer‐associated fibroblasts (paCAFs) Abbreviations: BM‐MSC, bone marrow‐derived mesenchymal stem cells; iCAF, inflammatory CAF; myCAF, myofibroblastic CAF; tr‐MSCF, tissue resident‐mesenchymal stem cell‐like fibroblasts; tr‐RF, tissue resident‐resting fibroblasts.

There were several drawbacks to this study. First, owing to the strict procedures for defining fibroblasts, we may have eliminated potential fibroblast populations. Second, as paCAFs were identified based on the expression of *PRRX1* and other CAF‐related markers, the results did not fully reflect the activation status of heterogeneous CAFs. However, we believe that our findings provide valuable insights for future studies.

## CONFLICT OF INTEREST

The authors declare that they have no competing interests.

## ETHICS APPROVAL AND CONSENT TO PARTICIPATE

This study was approved by the IRB at Asan Medical Center, Seoul.

## AUTHOR CONTRIBUTIONS

Chang Ohk Sung, Seok‐Hyung Kim, and Dakeun Lee conceived the project and provided leadership. Chang Ohk Sung and Dakeun Lee designed the study. Hee Chul Chung and Chang Ohk Sung analyzed the genomic data and interpreted the results. Dakeun Lee, Eun Jeong Cho, Hyeonjin Lee, Won‐Kyung Kim, Ji‐Hye Oh, and Seok‐Hyung Kim contributed to materials, analysis, and interpretation of the data. Dakeun Lee, Chang Ohk Sung, and Hee Chul Chung wrote the manuscript. All authors reviewed and approved the final manuscript.

## FUNDING INFORMATION

This study was supported by the Basic Science Research Program through the National Research Foundation of Korea (NRF), funded by the Ministry of Science, ICT & Future Planning (NRF‐2019R1A2C1084460 and NRF‐2021R1A2C2005853), the Bio and Medical Technology Development Program of the NRFK (NRF‐2019M3E5D4066900) of the Korean government, and grant 2021IP0013 from the Asan Institute for Life Sciences of Asan Medical Center, Korea.

## DATA AVAILABILITY STATEMENT

Count matrices and metadata for pan‐cancer scRNA‐seq data obtained from 36 patients are available at http://blueprint.lambrechtslab.org/. Processed scRNA‐seq data and metadata for 23 Korean patients with colorectal cancer are available in the NCBI Gene Expression Omnibus (GEO) database under the accession code GSE132465. Filtered stomach cancer scRNA‐seq data are available at https://dna‐discovery.stanford.edu/research/datasets/. Filtered BM‐MSC scRNA‐seq data are available in the GEO database under the accession code GSE147287.

## Supporting information

Supporting Information files
Supplementary Methods (PDF)

Figure S1 (PDF)

Figure S2 (PDF)

Figure S3 (PDF)

Figure S4 (PDF)

Figure S5 (PDF)

Figure S6 (PDF)

Figure S7 (PDF)

Figure S8 (PDF)

Table S1 (PDF)

## References

[1] Kalluri R . The biology and function of fibroblasts in cancer. Nat Rev Cancer. 2016;16:582–598. 27550820 10.1038/nrc.2016.73

[2] LeBleu VS , Kalluri R . A peek into cancer‐associated fibroblasts: origins, functions and translational impact. Dis model Mech. 2018;11:dmm029447. 29686035 10.1242/dmm.029447 PMC5963854

[3] Arina A , Idel C , Hyjek EM , et al. Tumor‐associated fibroblasts predominantly come from local and not circulating precursors. Proc Natl Acad Sci U S A. 2016;113:7551–7556. 27317748 10.1073/pnas.1600363113 PMC4941507

[4] Lee HO , Hong Y , Etlioglu HE , et al. Lineage‐dependent gene expression programs influence the immune landscape of colorectal cancer. Nat Genet. 2020;52:594–603. 32451460 10.1038/s41588-020-0636-z

[5] Qian J , Olbrecht S , Boeckx B , et al. A pan‐cancer blueprint of the heterogeneous tumor microenvironment revealed by single‐cell profiling. Cell Res. 2020;30:745–762. 32561858 10.1038/s41422-020-0355-0 PMC7608385

[6] Sathe A , Grimes SM , Lau BT , et al. Single‐cell genomic characterization reveals the cellular reprogramming of the gastric tumor microenvironment. J Clin Cancer Res. 2020;26:2640–2653. 10.1158/1078-0432.CCR-19-3231 PMC7269843 32060101

[7] Yeo SY , Lee KW , Shin D , An S , Cho KH , Kim SH . A positive feedback loop bi‐stably activates fibroblasts. Nat Commun. 2018;9:3016. 30069061 10.1038/s41467-018-05274-6 PMC6070563

[8] Sahai E , Astsaturov I , Cukierman E , et al. A framework for advancing our understanding of cancer‐associated fibroblasts. Nat Rev Cancer. 2020;20:174–186. 31980749 10.1038/s41568-019-0238-1 PMC7046529

[9] Öhlund D , Handly‐Santana A , Biffi G , et al. Distinct populations of inflammatory fibroblasts and myofibroblasts in pancreatic cancer. J Exp Med. 2017;214:579–596. 28232471 10.1084/jem.20162024 PMC5339682

[10] Wang Z , Li X , Yang J , et al. Single‐cell RNA sequencing deconvolutes the in vivo heterogeneity of human bone marrow‐derived mesenchymal stem cells. bioRxiv. 2020. 10.1101/2020.04.06.027904. PMC8579438 34803492

