# Supplementary material for: Integrated single‐cell RNA sequencing analyses suggest developmental paths of cancer‐associated fibroblasts with gene expression dynamics
Source: Clin Transl Med. 2021 Jul 19;11(7):e487. doi: 10.1002/ctm2.487 (PMC8287981; doi:10.1002/ctm2.487)
Supplement: Supplementary file 1 — Supporting Information files Supplementary Methods (PDF) [file CTM2-11-e487-s001.docx]

**Supplementary Methods**

**Single cell RNA sequencing (scRNA-seq) datasets**

The datasets used in this study are summarized as follows: (1) scRNA-seq raw gene expression matrices for colorectal (colon set 1), lung, ovarian, and breast cancers (http://blueprint.lambrechtslab.org/)^1^; (2) scRNA-seq gene expression data for colorectal cancer (GSE132465) (colon set 2)^2^; and (3) filtered scRNA-seq outputs for the stomach (https://dna-discovery.stanford.edu/research/datasets/).^3^ We divided the scRNA-seq data into tumor and normal data based on tissue origin.

**scRNA-seq dataset for bone marrow-derived mesenchymal stem cells (BM-MSCs)**
BM-MSC raw gene expression matrices of two different patients were retrieved from the NCBI Gene Expression Omnibus (GEO) database under the accession code GSE147287. We extracted data for approximately 1,500 cell lines randomly to balance the number of fibroblasts from colon set 1 for the analysis. The raw gene expression matrix was processed in the same manner with data processing of fibroblasts, and the BM-MSC processed data were combined with CRC1 data using the ComBat algorithm,^4^ as ComBat is one of the recommended methods for obtaining a batch-effect-corrected matrix for downstream analysis.^5^

**The Cancer Genome Atlas (TCGA) datasets**

Level 3 RNA-seq gene expression (Illumina_rnaseqv2-RSEM_genes_normalized) data were downloaded from TCGA for four solid tumor types, including colorectal adenocarcinoma (COADREAD), stomach adenocarcinoma (STAD), ovarian serous cystadenocarcinoma (OV), and lung cancer (LUAD and LUSC). The lung adenocarcinoma (LUAD) and lung squamous cell carcinoma (LUSC) datasets were analyzed independently. These data were preprocessed by the team at the Broad Institute (https://gdac.broadinstitute.org). Clinical data were retrieved from cBioPortal (https://www.cbioportal.org/).

**Preprocessing of scRNA-seq data**

Gene expression matrices were processed by applying two different criteria using the Seurat package (v3.2.0).^6,7^ Except for colon set 2 (GSE132465), which was already logTPM-normalized, we normalized scRNA-seq data with the NormalizeData function using the above-mentioned packages. For colon set 2 and stomach data, we applied the selection method reported by Lee et al.^2^ The mean expression criteria ranged from 0.0125 to 3, and using the FindVariableGenes function, a dispersion of more than 0.5 was performed using selected variably expressed genes for selecting variable features. PCA was performed with the selected variably expressed genes using the RunPCA function. Subsequently, we constructed a k-nearest neighbor graph based on the Euclidean distance in the PCA space using the FindNeighbors function. We applied the Louvain algorithm with a resolution of 0.8 to iteratively group cells using the FindClusters function. Lastly, we generated t-SNEs with clusters using the RunTSNE function. For pan-cancer data, including those from colon set 1, lung, ovarian, and breast cancer datasets, we applied the filtering method reported by Qian et al.^1^ Cells with < 401 unique molecular identifiers (UMIs), > 6,000 or < 201 expressed genes, and > 25% of the reads mapping to mitochondrial RNA, were removed. Mean expression between 0.125 and 3 and a dispersion of more than 0.5 were selected using the FindVariableGenes function. Thereafter, the following confounding factors were regressed using the ScaleData function: cell cycle scores output by the CellCycleScoring function, percentage of mitochondrial DNA, patient ID, and number of UMIs. The subsequent methods for cluster calculation were the same as those used for colon set 2 and stomach data.

**Identification of fibroblasts from processed scRNA-seq gene expression data**

We used two different methods independently and extracted the data on fibroblasts. First, we used known fibroblast marker genes (*COL1A1*, *BGN*, and *DCN*)^1^ and the FeaturePlot function for t-SNE data, to identify fibroblasts. Second, we used the SingleR package (v1.3.7)^8^ for the original tumor and normal matrices directly to extract the data on fibroblasts. Based on these two independent outputs, we defined overlapping cells as fibroblasts in each sample and used them for the subsequent analyses. These stringent approaches for the selection of fibroblasts reduced the number of fibroblasts; however, we adopted the two-step approach to isolate fibroblasts in a more stringent manner, as cancer-associated fibroblasts (CAFs) are highly heterogeneous and lack specific markers.

**Reclustering of fibroblasts for identification of subgroups**

We applied k-means clustering using the k-means function for each set of fibroblast data. The optimal number of clusters for each sample was determined based on the sum of squared error for each sample analysis, and principal component analysis (PCA) was performed to visualize the clusters. Subsequently, we analyzed *PRRX1* expression^9,10^ to determine the activation status of CAFs in each cluster. Furthermore, we explored the expression levels of known CAF-related genes^11,12^ in each cluster. In this study, we referred to the group separated after initial k-means clustering analyses as “cluster”. The subcluster (subgroup) was indicated when a single cluster was segregated into further subgroups after subgroup analyses. When we performed subgroup analysis, we applied the same method used for k-means clustering, and this was performed in perpetually activated (pa)CAF groups of colorectal and lung cancer datasets.

**Identification of distinct CAF subtypes**

Specific genes, identified in a previous study,^13^ were used to identify the panCAF (*COL1A1*, *FAP*, *PDPN*, and *DCNVIM*), myCAF (*ACTA2*, *TAGL*, *MMP11*, *MYL9*, *HOPX*, *POSTN*, *TPM1*, and *TPM2*), and iCAF (*IL6*, *PDGFRA*, *CXCL12*, *CFD, DPT*, *LMNA*, *AGTR1*, *HAS1*, *CXCL1*, *CXCL2*, *CCL2*, and *IL8*) subtypes for the defined fibroblasts. Each signature was determined by considering the mean expression of genes. We compared the gene expression of *myCAF* and *iCAF* in CAF clusters.

**Identification of suggested paths in fibroblasts between tumor and normal tissues**

Spearman’s correlation analysis was performed for all fibroblasts between tumor and normal tissues to identify the fibroblasts in normal tissue that exhibited the best correlation with CAFs in terms of global gene expression. Thereafter, we determined the cluster belonging to the fibroblasts in normal tissues with the highest correlation for CAFs. The correlation results were visualized using the ggalluvial package (v0.12.2).^14^ The same analysis was also performed for BM-MSCs and fibroblast groups [tr-MSCF (tissue-resident mesenchymal stem cell-like fibroblast) and paCAF groups] of colon set 1. Subsequently, we used the limma R package (v3.45.10)^15^ to identify DEGs among the paCAF groups and normal fibroblast (NB) groups including tr-MSCF and tr-RF (tissue resident-resting fibroblast) groups. An adjusted p-value (FDR q) < 0.01 was used to filter the data on genes, and we used the EnhancedVolcano R package (v1.7.16) for filtered data on genes to extract and visualize data on final DEGs for each sample. Additionally, we used the monocle^16^ R package (v0.2.3.0) and slingshot^17^ R package (v1.8.0) for conducting trajectory analysis to determine the pseudotime and lineage in fibroblast clusters. We further performed pseudotime analysis using RNA velocity^18^ with the raw scRNA-seq data of colon set 1 obtained from the ArrayExpress under the accession codes E-MTAB-8410. The initial raw data were processed using the CellRanger 4.0.0 pipeline (10X Genomics) and velocyto Python package (v.0.17.16). Generated loom files were converted using Seurat and SeuratWrappers R package (v.0.3.0) to Seurat objects and merged, followed by filtering with the same criteria used at the scRNA-seq preprocessing stage as mentioned above. Subsequently, we used SeuratDisk R package (0.0.0.9019) to convert the result to h5ad file followed by scVelo Python package (v0.2.3) to perform RNA velocity analysis.

**Gene set for CAFs and clinical significance**

We defined the paCAF signature gene set for colorectal cancer based on the common DEGs (paCAF group vs. tr-MSCF group) from scRNA-seq data of CRC 1 and CRC 2 sets. Additionally, we defined myCAF signature gene set with the same datasets (Common DEGs between myCAF group and tr-MSCF group of the two colon sets). Subsequently, we performed GSVA v1.24.2^19^ for the newly defined paCAF and myCAF signature gene sets in TCGA CRC RNA sequencing data to identify each signature gene set enrichment score of individual TCGA CRC cancer tissue. TCGA samples were classified using the median paCAF signature score obtained from GSVA, and survival analysis was performed using the survival R package (v3.2-7) to identify the clinical significance of paCAF signature. In the case of myCAF enrichment scores, we performed a Spearman correlation test to investigate the correlation between the score levels and the disease stages in colorectal cancer. We applied the same processes, except obtaining myCAF signature gene set, to the remaining tumor sets. Additionally, we divided colon TCGA CRC RNA sequencing data into four Consensus Molecular Subgroup (CMS)^20^ using the “CMSclassifier” package.

**Statistical analysis**

Correlation analysis of continuous variables was performed using Spearman’s correlation analysis. According to the normality of data distribution, the Wilcoxon rank-sum test (nonparametric) or Student’s *t*-test (parametric) was used to evaluate the significance of differences among continuous variables between the two groups. The log-rank test and multivariate Cox proportional hazards regression analyses were also performed. All statistical analyses were performed using R version 3.5.2 (R Foundation for Statistical Computing, Vienna, Austria).

**References**

1 Qian, J., Olbrecht, S., Boeckx, B. *et al.* A pan-cancer blueprint of the heterogeneous tumor microenvironment revealed by single-cell profiling. *Cell research* **30**, 745-762, doi:10.1038/s41422-020-0355-0 (2020).

2 Lee, H. O., Hong, Y., Etlioglu, H. E. *et al.* Lineage-dependent gene expression programs influence the immune landscape of colorectal cancer. *Nature genetics* **52**, 594-603, doi:10.1038/s41588-020-0636-z (2020).

3 Sathe, A., Grimes, S. M., Lau, B. T. *et al.* Single-Cell Genomic Characterization Reveals the Cellular Reprogramming of the Gastric Tumor Microenvironment. *Clinical cancer research : an official journal of the American Association for Cancer Research* **26**, 2640-2653, doi:10.1158/1078-0432.Ccr-19-3231 (2020).

4 Akiyama, K., You, Y. O., Yamaza, T. *et al.* Characterization of bone marrow derived mesenchymal stem cells in suspension. *Stem cell research & therapy* **3**, 40, doi:10.1186/scrt131 (2012).

5 Tran, H. T. N., Ang, K. S., Chevrier, M. *et al.* A benchmark of batch-effect correction methods for single-cell RNA sequencing data. *Genome biology* **21**, 12, doi:10.1186/s13059-019-1850-9 (2020).

6 Butler, A., Hoffman, P., Smibert, P., Papalexi, E. & Satija, R. Integrating single-cell transcriptomic data across different conditions, technologies, and species. *Nature Biotechnology* **36**, 411-420, doi:10.1038/nbt.4096 (2018).

7 Stuart, T., Butler, A., Hoffman, P. *et al.* Comprehensive Integration of Single-Cell Data. *Cell* **177**, 1888-1902.e1821, doi:10.1016/j.cell.2019.05.031 (2019).

8 Aran, D., Looney, A. P., Liu, L. *et al.* Reference-based analysis of lung single-cell sequencing reveals a transitional profibrotic macrophage. *Nature Immunology* **20**, 163-172, doi:10.1038/s41590-018-0276-y (2019).

9 Tomaru, Y., Hasegawa, R., Suzuki, T. *et al.* A transient disruption of fibroblastic transcriptional regulatory network facilitates trans-differentiation. *Nucleic acids research* **42**, 8905-8913, doi:10.1093/nar/gku567 (2014).

10 Yeo, S. Y., Lee, K. W., Shin, D., An, S., Cho, K. H. & Kim, S. H. A positive feedback loop bi-stably activates fibroblasts. *Nature communications* **9**, 3016, doi:10.1038/s41467-018-05274-6 (2018).

11 LeBleu, V. S. & Kalluri, R. A peek into cancer-associated fibroblasts: origins, functions and translational impact. *Disease models & mechanisms* **11**, doi:10.1242/dmm.029447 (2018).

12 Elyada, E., Bolisetty, M., Laise, P. *et al.* Cross-Species Single-Cell Analysis of Pancreatic Ductal Adenocarcinoma Reveals Antigen-Presenting Cancer-Associated Fibroblasts. *Cancer Discov* **9**, 1102-1123, doi:10.1158/2159-8290.Cd-19-0094 (2019).

13 Elyada, E., Bolisetty, M., Laise, P. *et al.* Cross-Species Single-Cell Analysis of Pancreatic Ductal Adenocarcinoma Reveals Antigen-Presenting Cancer-Associated Fibroblasts. **9**, 1102-1123, doi:10.1158/2159-8290.CD-19-0094 %J Cancer Discovery (2019).

14 Brunson, J. C. J. J. o. O. S. S. ggalluvial: Layered Grammar for Alluvial Plots. **5**, 2017 (2020).

15 Ritchie, M. E., Phipson, B., Wu, D. *et al.* limma powers differential expression analyses for RNA-sequencing and microarray studies. *Nucleic acids research* **43**, e47-e47, doi:10.1093/nar/gkv007 %J Nucleic Acids Research (2015).

16 Qiu, X., Hill, A., Packer, J., Lin, D., Ma, Y. A. & Trapnell, C. Single-cell mRNA quantification and differential analysis with Census. *Nature methods* **14**, 309-315, doi:10.1038/nmeth.4150 (2017).

17 Street, K., Risso, D., Fletcher, R. B. *et al.* Slingshot: cell lineage and pseudotime inference for single-cell transcriptomics. *BMC genomics* **19**, 477, doi:10.1186/s12864-018-4772-0 (2018).

18 La Manno, G., Soldatov, R., Zeisel, A. *et al.* RNA velocity of single cells. *Nature* **560**, 494-498, doi:10.1038/s41586-018-0414-6 (2018).

19 Hanzelmann, S., Castelo, R. & Guinney, J. GSVA: gene set variation analysis for microarray and RNA-seq data. *BMC bioinformatics* **14**, 7, doi:10.1186/1471-2105-14-7 (2013).

20 Guinney, J., Dienstmann, R., Wang, X. *et al.* The consensus molecular subtypes of colorectal cancer. *Nat Med* **21**, 1350-1356, doi:10.1038/nm.3967 (2015).
