# Supplementary material for: Integrated single‐cell RNA sequencing analyses suggest developmental paths of cancer‐associated fibroblasts with gene expression dynamics
Source: Clin Transl Med. 2021 Jul 19;11(7):e487. doi: 10.1002/ctm2.487 (PMC8287981; doi:10.1002/ctm2.487)
Supplement: Supplementary file 2 — Figure S1 (PDF) [file CTM2-11-e487-s004.pdf]

Figure S1

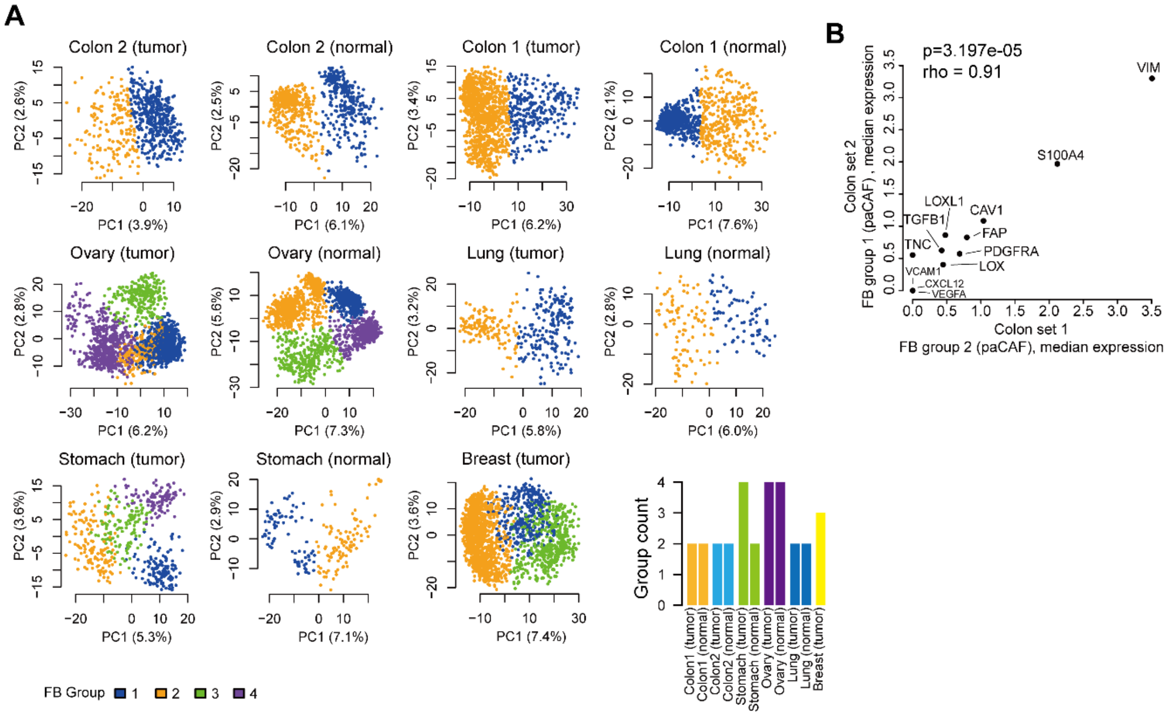

Figure S1. A, Clusters of cancer-associated fibroblasts (CAFs) and normal fibroblasts (NFs) in various organs, identified using k-means clustering. **B**, The paCAF groups from two different colon sets showed significant correlation with CAF-related gene expression (Spearman correlation test). paCAF, perpetually activated cancer-associated fibroblast.
