# Supplementary material for: Integrated single‐cell RNA sequencing analyses suggest developmental paths of cancer‐associated fibroblasts with gene expression dynamics
Source: Clin Transl Med. 2021 Jul 19;11(7):e487. doi: 10.1002/ctm2.487 (PMC8287981; doi:10.1002/ctm2.487)
Supplement: Supplementary file 3 — Figure S2 (PDF) [file CTM2-11-e487-s009.pdf]

Figure S2

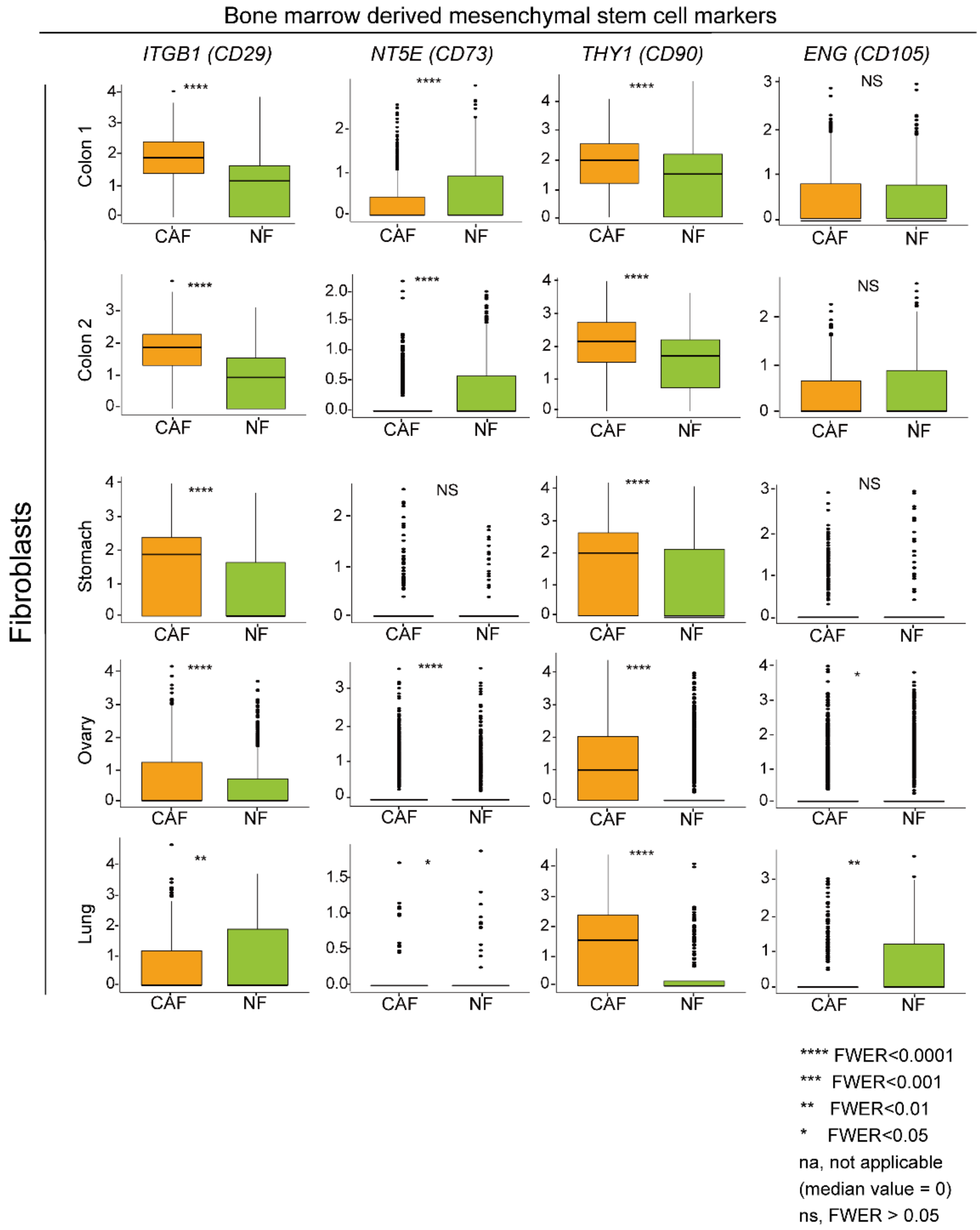

Figure S2. Expression levels of bone marrow-derived mesenchymal stem cell markers of fibroblasts in cancer tissues (CAF) and normal tissues (NF) from various organs. CAF, cancer-associated fibroblast; NF, normal fibroblast.
