# Supplementary material for: Integrated single‐cell RNA sequencing analyses suggest developmental paths of cancer‐associated fibroblasts with gene expression dynamics
Source: Clin Transl Med. 2021 Jul 19;11(7):e487. doi: 10.1002/ctm2.487 (PMC8287981; doi:10.1002/ctm2.487)
Supplement: Supplementary file 4 — Figure S3 (PDF) [file CTM2-11-e487-s008.pdf]

Figure S3

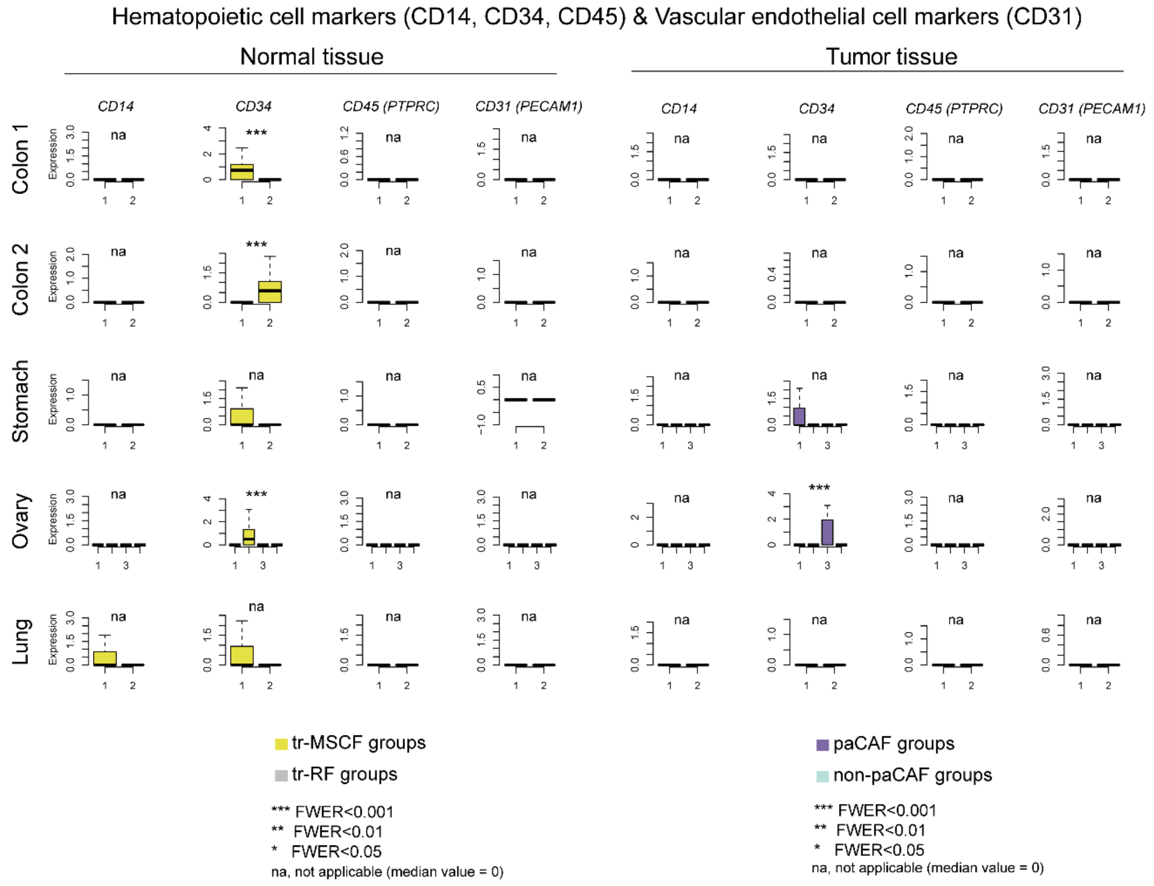

Figure S3. Expression of hematopoietic stem cell or vascular endothelial cell (CD31) markers in cancer-associated fibroblasts (CAFs) and normal fibroblasts (NFs) from various organs. Lack of expression of hematopoietic cell markers (CD14 and CD45) and vascular endothelial cell marker *PECAM-1* (CD31) indicated that the cells were derived from MSCs. Although CD34 has been considered a negative BM-MSC marker for decades, several previous studies demonstrated that a fraction of tr-MSCs, and not cultured MSCs, can express CD34.[1,2] In line with this, some paCAF clusters and normal fibroblast groups showed CD34 expression. BM-MSC, bone marrow-mesenchymal stem cell; tr-MSCF, tissue resident mesenchymal stem cell-like fibroblast; tr-RF, tissue resident-resting fibroblast; paCAF, perpetually activated CAF.

1 Akiyama, K., You, Y. O., Yamaza, T. *et al.* Characterization of bone marrow derived mesenchymal stem cells in suspension. *Stem cell research & therapy* **3**, 40, doi:10.1186/scrt131 (2012).

2 Lin, C. S., Ning, H., Lin, G. & Lue, T. F. Is CD34 truly a negative marker for mesenchymal stromal cells? *Cytotherapy* **14**, 1159-1163, doi:10.3109/14653249.2012.729817 (2012).
