# Supplementary material for: Integrated single‐cell RNA sequencing analyses suggest developmental paths of cancer‐associated fibroblasts with gene expression dynamics
Source: Clin Transl Med. 2021 Jul 19;11(7):e487. doi: 10.1002/ctm2.487 (PMC8287981; doi:10.1002/ctm2.487)
Supplement: Supplementary file 5 — Figure S4 (PDF) [file CTM2-11-e487-s007.pdf]

Figure S4

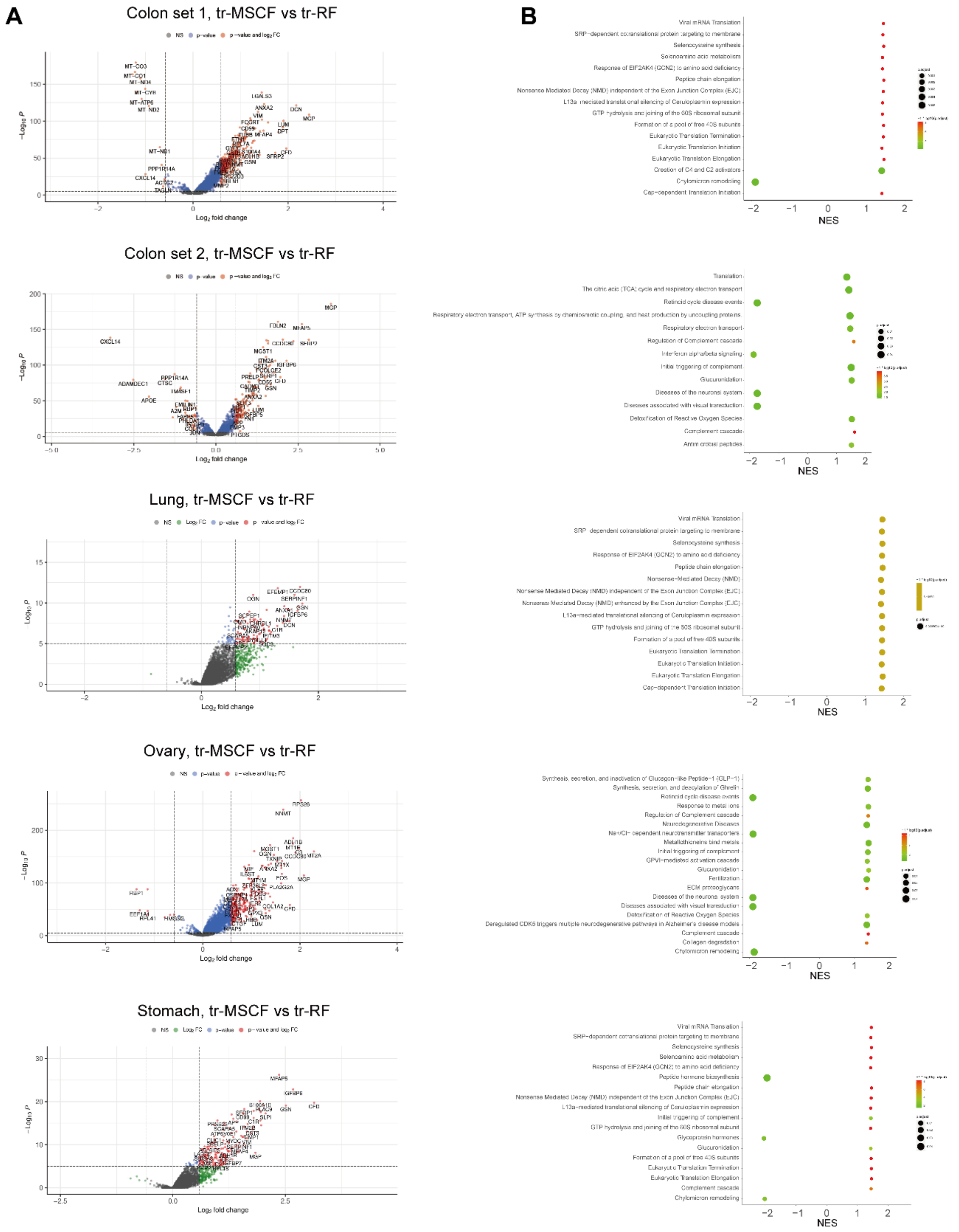

Figure S4. Differentially expressed genes (DEGs) between tr-MSCFs and tr-RFs in various organs. Most of the DEGs were genes upregulated in tr-MSCFs. These findings suggest that tr-MSCFs are biologically active, whereas tr-RFs are transcriptionally less active. tr-MSCF, tissue resident mesenchymal stem cell-like fibroblast; tr-RF, tissue resident-resting fibroblast.
