# Supplementary material for: Integrated single‐cell RNA sequencing analyses suggest developmental paths of cancer‐associated fibroblasts with gene expression dynamics
Source: Clin Transl Med. 2021 Jul 19;11(7):e487. doi: 10.1002/ctm2.487 (PMC8287981; doi:10.1002/ctm2.487)
Supplement: Supplementary file 6 — Figure S5 (PDF) [file CTM2-11-e487-s010.pdf]

Figure S5

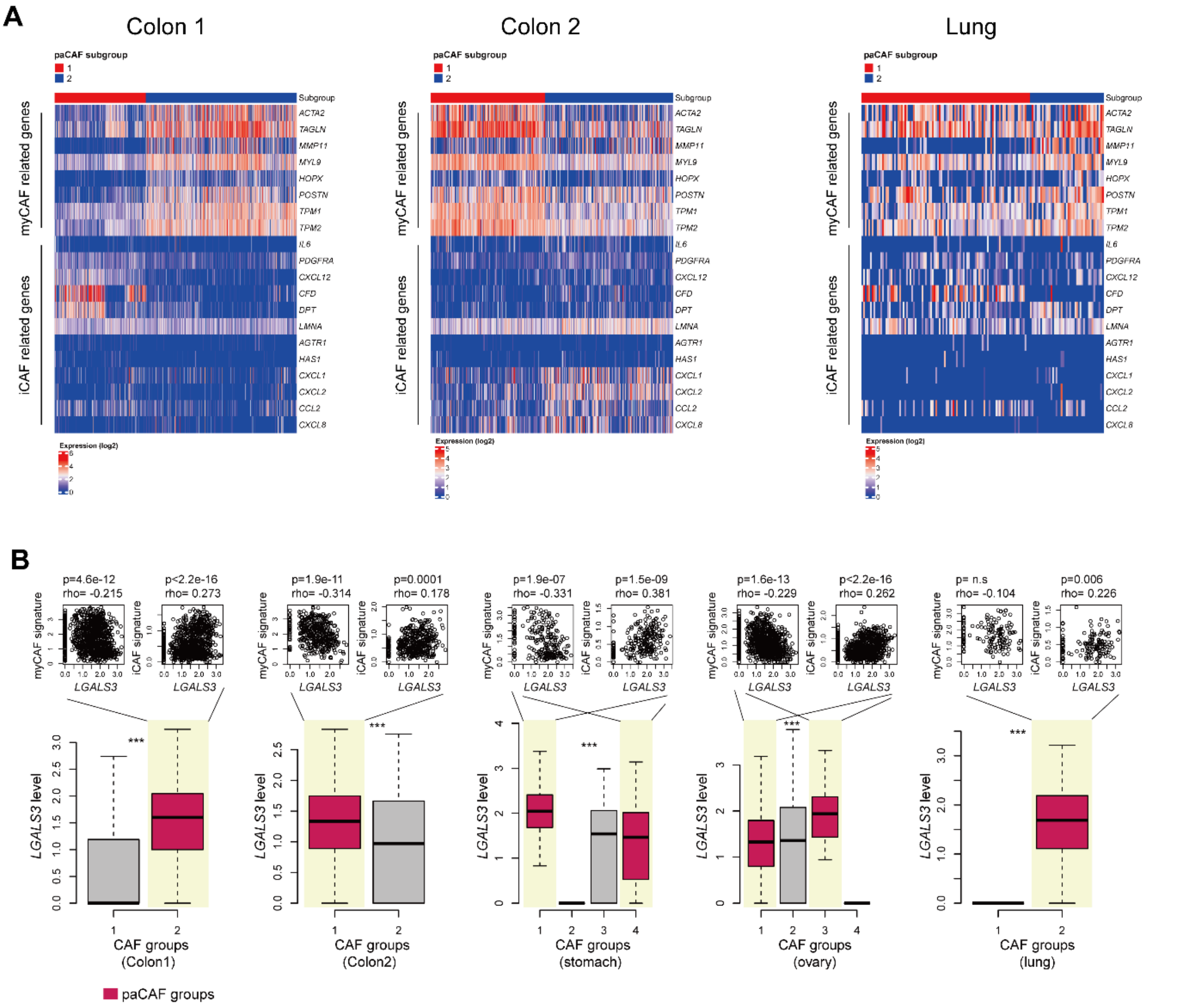

Figure S5. A, myCAF- and iCAF-related gene expression in subgroups of the paCAF group from colorectal and lung cancers. **B,** *LGALS3* expression according to the CAF groups and correlation with myCAF and iCAF signatures (Spearman correlation test). CAF, cancer-associated fibroblast; myCAF, myofibroblastic CAF; iCAF, inflammatory CAF; paCAF, perpetually activated CAF.
