# Supplementary material for: Integrated single‐cell RNA sequencing analyses suggest developmental paths of cancer‐associated fibroblasts with gene expression dynamics
Source: Clin Transl Med. 2021 Jul 19;11(7):e487. doi: 10.1002/ctm2.487 (PMC8287981; doi:10.1002/ctm2.487)
Supplement: Supplementary file 7 — Figure S6 (PDF) [file CTM2-11-e487-s003.pdf]

Figure S6

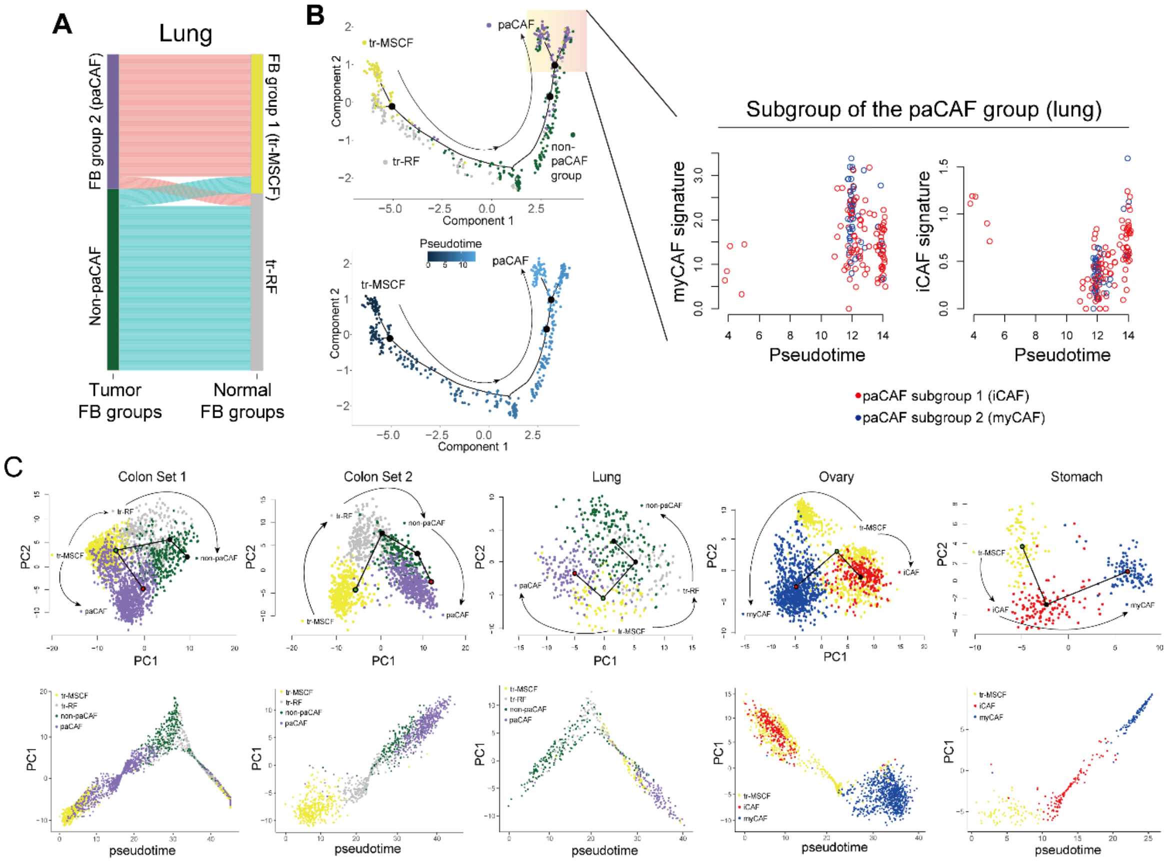

Figure S6. A, Spearman correlation analysis of CAFs and NFs in the lung dataset. **B,** Trajectory and pseudotime analyses on different subgroups of CAFs and tr-MSCFs in lung tissues. **C,** Validation of trajectory and pseudotime analyses for each sample using the slingshot R package. CAF, cancer-associated fibroblast; NF, normal fibroblast; tr-MSCF, tissue resident mesenchymal stem cell-like fibroblast.
