# Supplementary material for: Integrated single‐cell RNA sequencing analyses suggest developmental paths of cancer‐associated fibroblasts with gene expression dynamics
Source: Clin Transl Med. 2021 Jul 19;11(7):e487. doi: 10.1002/ctm2.487 (PMC8287981; doi:10.1002/ctm2.487)
Supplement: Supplementary file 8 — Figure S7 (PDF) [file CTM2-11-e487-s006.pdf]

Figure S7

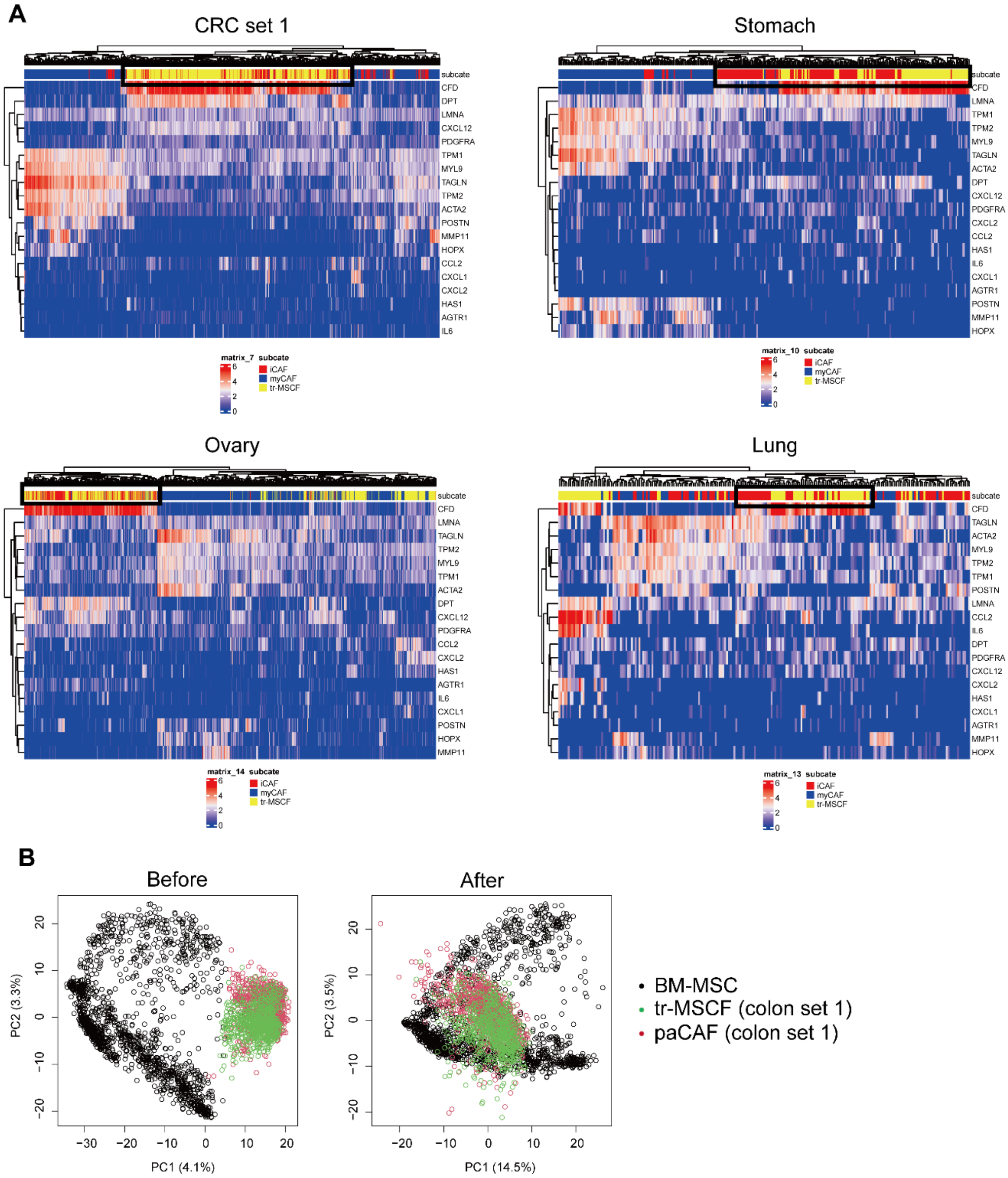

Figure S7. A, Unsupervised clustering shows the tendency of tr-MSCFs and iCAFs for clustering. **B,** Combined BM-MSC data and colon set 1 data, obtained using ComBat, for downstream analysis. tr-MSCF, tissue resident mesenchymal stem cell-like fibroblast; BM-MSC, bone marrow-mesenchymal stem cell; CAF, cancer associated fibroblast; iCAF, inflammatory CAF; paCAF, perpetually activated CAF.
