# Supplementary material for: Integrated single‐cell RNA sequencing analyses suggest developmental paths of cancer‐associated fibroblasts with gene expression dynamics
Source: Clin Transl Med. 2021 Jul 19;11(7):e487. doi: 10.1002/ctm2.487 (PMC8287981; doi:10.1002/ctm2.487)
Supplement: Supplementary file 9 — Figure S8 (PDF) [file CTM2-11-e487-s005.pdf]

Figure S8

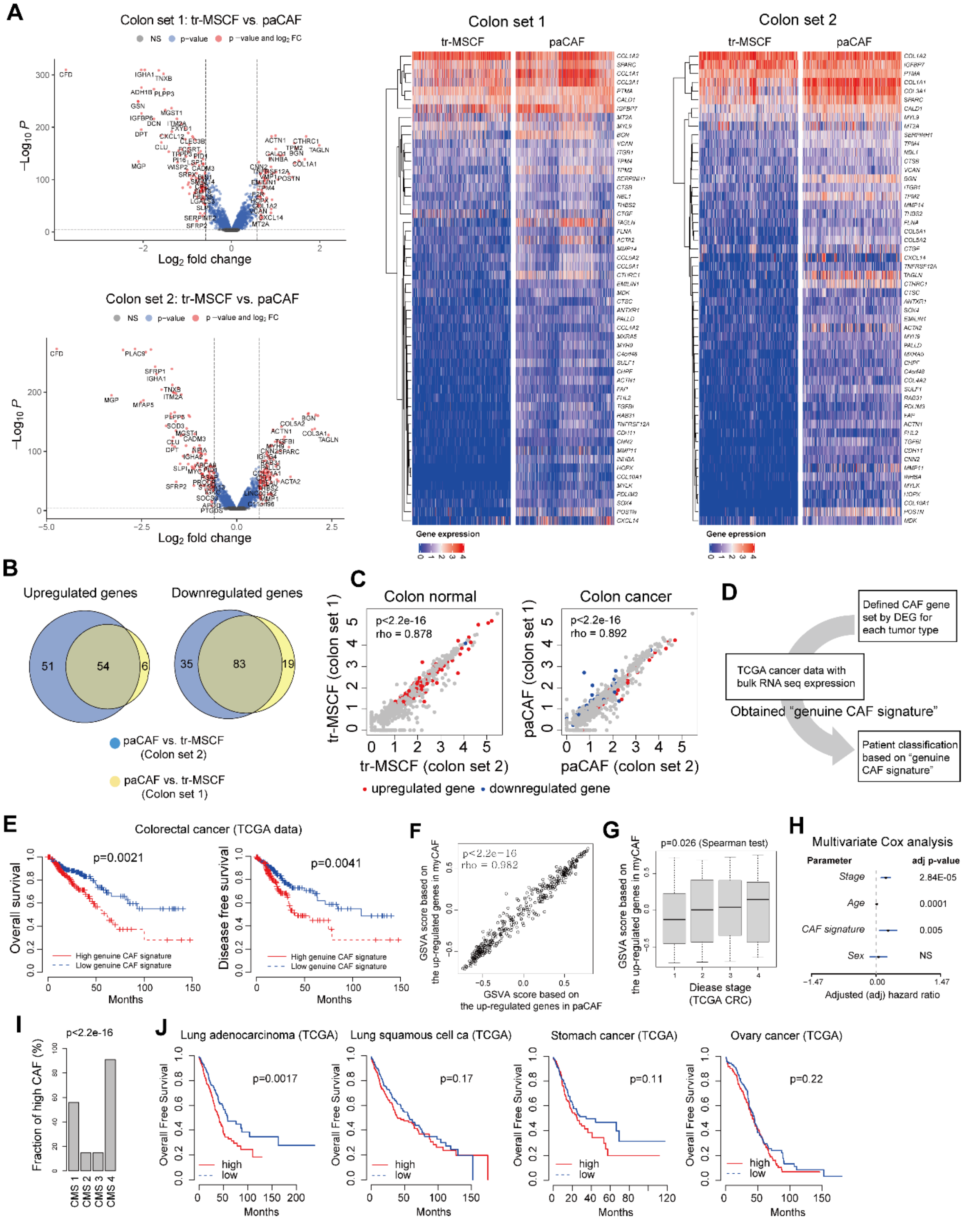

Figure S8. **A,** Differentially expressed genes (DEGs) between paCAF and tr-MSCF from two colorectal data sets. **B,** Overlap of significant genes between two colorectal data sets. **C,** Correlation of global gene expression of paCAF and tr-MSCF between two colorectal data sets. **D,** Defining the CAF signature gene set based on DEGs, and classification of patients based on the CAF signature gene set score (genuine CAF signature) from GSVA analysis. **E,** Overall

survival of patients with colorectal cancer according to the CAF signature gene set score (GSVA score) by median value (Log-rank test). **F,** We further identified that the CAF signature gene set score was strongly associated with the myCAF signature gene set score in colorectal cancer, as determined using DEGs between myCAFs and tr-MSCFs from two colorectal data sets. **G-H,** Level of the myCAF signature gene set score in colorectal cancer was associated with the disease stage in colorectal cancer (G); however, the clinical significance of the CAF signature gene set score was independent of the disease stage as well as age (adjusted p value by Multivariate Cox regression analysis) (H). **I,** Correlation between high genuine CAF signature and CMS classification in colorectal cancer (Fisher's exact test). **J,** Overall survival of patients according to genuine CAF signature score obtained from each tumor type in the same manner for each tumor as that in colorectal cancer (Log-rank test). CAF, cancer associated fibroblast; paCAF, perpetually activated CAF; myCAF, myofibroblastic CAF; GSVA, gene set variation analysis; tr-MSCF, tissue resident-mesenchymal stem cell like fibroblast; CMS, Consensus Molecular Subgroups.
