# Supplementary material for: Integrated single‐cell RNA sequencing analyses suggest developmental paths of cancer‐associated fibroblasts with gene expression dynamics
Source: Clin Transl Med. 2021 Jul 19;11(7):e487. doi: 10.1002/ctm2.487 (PMC8287981; doi:10.1002/ctm2.487)
Supplement: Supplementary file 10 — Table S1 (PDF) [file CTM2-11-e487-s002.pdf]

Supplementary Table 1. Significantly upregulated genes in paCAFs of colorectal cancers driven from single-cell RNA sequencing data.

	Commonly upregulated genes in paCAF of two colon sets
Gene lists	BGN, TPM2, CTHRC1, POSTN, COL5A2, ACTN1, COL3A1, CDH11, COL1A1, TAGLN, TFGBI, SULF1, EMILIN1, MYH9, INHBA, TPM4, CALD1, CNN2, SPARC, COL5A1, SERPINH1, C4orf48, PTMA, RAB31, ITGB1, PALLD, MYLK, MXRA5, COL4A2, FAP, CTSB, MDK, NBL1, SOX4, MMP14, COL10A1, ACTA2, FLNA, ANTXR1, CHPF, FHL2, MMP11, MYL9, HOPX, COL1A2, PDLIM3, THBS2, CTSC, TNFRSF12A, VCAN, CTGF, IGFBP7, MT2A, CXCL14
